# Exploring the Efficacy of Replacing Linear Paper-Based Patient Cases in Problem-Based Learning With Dynamic Web-Based Virtual Patients: Randomized Controlled Trial

**DOI:** 10.2196/jmir.3748

**Published:** 2014-11-05

**Authors:** Terry Poulton, Rachel H Ellaway, Jonathan Round, Trupti Jivram, Sheetal Kavia, Sean Hilton

**Affiliations:** ^1^Institute of Medical and Biomedical EducationSt George's, University of LondonLondonUnited Kingdom; ^2^Human SciencesNorthern Ontario School of MedicineOntario, ONCanada; ^3^University of NicosiaNicosiaCyprus

**Keywords:** problem-based learning, decision making, education, medical, virtual patients, curriculum

## Abstract

**Background:**

Problem-based learning (PBL) is well established in medical education and beyond, and continues to be developed and explored. Challenges include how to connect the somewhat abstract nature of classroom-based PBL with clinical practice and how to maintain learner engagement in the process of PBL over time.

**Objective:**

A study was conducted to investigate the efficacy of decision-PBL (D-PBL), a variant form of PBL that replaces linear PBL cases with virtual patients. These Web-based interactive cases provided learners with a series of patient management pathways. Learners were encouraged to consider and discuss courses of action, take their chosen management pathway, and experience the consequences of their decisions. A Web-based application was essential to allow scenarios to respond dynamically to learners’ decisions, to deliver the scenarios to multiple PBL classrooms in the same timeframe, and to record centrally the paths taken by the PBL groups.

**Methods:**

A randomized controlled trial in crossover design was run involving all learners (N=81) in the second year of the graduate entry stream for the undergraduate medicine program at St George’s University of London. Learners were randomized to study groups; half engaged in a D-PBL activity whereas the other half had a traditional linear PBL activity on the same subject material. Groups alternated D-PBL and linear PBL over the semester. The measure was mean cohort performance on specific face-to-face exam questions at the end of the semester.

**Results:**

D-PBL groups performed better than linear PBL groups on questions related to D-PBL with the difference being statistically significant for all questions. Differences between the exam performances of the 2 groups were not statistically significant for the questions not related to D-PBL. The effect sizes for D-PBL–related questions were large and positive (>0.6) except for 1 question that showed a medium positive effect size. The effect sizes for questions not related to D-PBL were all small (≤0.3) with a mix of positive and negative values.

**Conclusions:**

The efficacy of D-PBL was indicated by improved exam performance for learners who had D-PBL compared to those who had linear PBL. This suggests that the use of D-PBL leads to better midterm learning outcomes than linear PBL, at least for learners with prior experience with linear PBL. On the basis of tutor and student feedback, St George’s University of London and the University of Nicosia, Cyprus have replaced paper PBL cases for midstage undergraduate teaching with D-PBL virtual patients, and 6 more institutions in the ePBLnet partnership will be implementing D-PBL in Autumn 2015.

##  Introduction

### Overview

The early years of medical training have seen a progressive move away from curricula organized around single-discipline bioscience material and the use of passive forms of instruction [1]. Problem-based learning (PBL) has been widely adopted as a way of integrating knowledge acquisition, teamwork, and problem-solving skills using active learning techniques within small-group settings [2]. However, PBL has remained relatively unchanged since it was introduced with paper cases—the principal medium around which PBL activities are organized. Even the use of the Internet has, at least so far, had limited impact on the design of the PBL case or the activities that are structured around it.

There are a number of educational technologies that share certain characteristics with PBL. For instance, virtual patients are on-screen learning resources that typically present a clinical problem for learners to solve or manage, and in doing so involve aspects of both PBL and simulation [3]. Although virtual patients can come in many forms, one of the more common forms is the branched case where learners select the best available course of action from predefined options [4]; each decision the learner makes can have consequences and lead to different outcomes.

St George’s University of London (SGUL) replaced paper PBL cases for midstage undergraduate teaching with decision-PBL (D-PBL) cases. These are Web-based branching virtual patients that require learners to discuss, debate, and make patient management decisions to negotiate the case [5]. This paper describes the efficacy of this approach using results from a controlled trial comparing end-of-year face-to-face exam performance for learners in PBL groups who worked with linear or branched cases. The paper closes with a consideration of the implications of this approach to teaching tomorrow’s doctors.

### Background

PBL involves a combination of students working in small groups with a facilitator and independent research. Each group works through a predefined patient case, discussing the information provided and implied, exploring possible diagnoses, suggesting investigations and treatments, and identifying the research they need to undertake to be able to resolve the case [6]. Learners then undertake their research before the next face-to-face session. The case may be resolved in the second face-to-face session or a second round of research may be identified which is then resolved in a third and final session. Groups of learners are typically given 1 PBL case per week. The pattern is repeated each week with different cases over a semester, term, or year. Although each PBL group will approach a case in different ways, the group facilitator directs the proceedings using the predefined PBL case outline (that the learners do not see) that sets out the key issues, anticipated learning objectives, and other intended features of the case, that allows them to keep learners from straying too far from the intended learning outcomes for each case.

PBL has been deployed in many different contexts [7] and although the efficacy and effectiveness of PBL continues to be debated [8-11], research into PBL indicates that its benefits come from key components of the PBL activity that tend to be obscured if PBL is considered as a single indivisible intervention [12,13]. These components include the construction of the PBL case, the behavior of the facilitator, and the curriculum context for the activity [14-16]. A key limitation of using paper-based cases is that they can only unfold in a single direction giving learners little or no opportunity to influence the outcome of the case.

There are other teaching modalities in medical education that employ cases, including simulation, case-based learning, and virtual patients, reflecting the role of the patient case as “the primary, vicarious means of shaping clinical judgment for new learners and experienced practitioners alike” [17]. Simulation differs from PBL in that the former is directed more to skills training and teamwork, but there are also similarities [18,19]. Virtual patients are interactive computer simulations of “real-life clinical scenarios for the purpose of medical training, education, or assessment” [20]. Although virtual patients can take many forms, they are intended usually for individual study [4]. The use of virtual patients for PBL has not previously been the focus of PBL developers although a number of schools have placed their paper-based PBL cases online as static documents [21].

The study presented here explored the efficacy of D-PBL in terms of student exam performance. Our working hypothesis was that D-PBL experiences would lead to better exam performance than linear PBL experiences. A randomized controlled trial in crossover design was run to compare aggregate learner performance in those learners who had D-PBL with those who had linear PBL with supplementary PBL “triggers” to compensate for the absence of D-PBL. The trial analyzed students’ performance on questions related to D-PBL decision points compared with their performance on questions not related to D-PBL. For each comparison cohort the learning activity was different, but the learning objectives were the same.

## Methods

### Study Context

SGUL is located in South London in the United Kingdom and runs a multiple stream medical education program. The undergraduate entry stream undertake a 5-year program and the graduate entry stream undertake a 4-year program. SGUL has employed PBL since the 1990s [21] and at the time of this study the 140 graduate entry learners (who were the participants in this study) completed 2 years of full PBL and then completed the 2 remaining years of clinical attachments.

A project was initiated in 2007 to rewrite the second year graduate entry paper PBL cases as branching virtual patients (D-PBL) and to modify the facilitation model to stimulate debate around predefined choices, and their consequences, at key points in the D-PBL virtual patient case [5]. The objective was to make PBL more engaging for learners with prior PBL experience by adding structured options and alternative outcomes to the linear PBL activity model, and encouraging robust debate around structured patient management options and the consequences of pursuing a particular course of action. This was called decision-PBL (D-PBL).

### Study Intervention

A D-PBL virtual patient case is designed as a series of interconnected nodes, each of which is presented as a Web page. Each node represents a step in the unfolding of the case. Some nodes are connected in chains; others have multiple nodes linked to them that allow learners to choose which path they will follow. Learners can only take 1 path through a case and they need to deal with the consequences of their decisions as the D-PBL case unfolds. The small-group facilitator encourages learners to consider the options and to debate different courses of action at each D-PBL decision point. Tutors report increased student engagement at option points and students express the value of simulating the experience of managing cases in real life, including the stresses that can lead to poor decisions [22,23].

The use of a Web-based virtual patient delivery system was essential to creating and running D-PBL activities. The virtual patient cases were to be delivered to multiple PBL classrooms in the same time frame, the scenarios were required to respond to the choices of different learner groups dynamically and independently, and the paths taken by the individual groups were tracked and logged centrally. The latter would permit later analysis and research into the paths that groups took to better understand the effectiveness of the option steps and to iteratively improve the cases.

To minimize the impact on the curriculum as a whole, D-PBL was designed to only differ from traditional linear PBL in a few key areas. D-PBL (like PBL) involved small-group (6 to 8) learners, face-to-face sessions at the start and end of the week, independent research and study between face-to-face sessions, scaffolding by a case with triggers based around problems, and 1 case worked on per week. The differences were the replacement of static paper cases with dynamic Web-based virtual patient cases and the periodic debates and decision making when alternative paths were presented (within face-to-face sessions).

The 5-week Life Protection module (covering immunology, pathology, hematology, and microbiology material) in the second-year SGUL graduate entry stream was selected as the context for the study. All 5 cases within the Life Protection module were rewritten both as linear PBL cases and as D-PBL cases using the same virtual patient software; the only difference being the addition of different routes through the case for the D-PBL versions.

**Figure 1:**
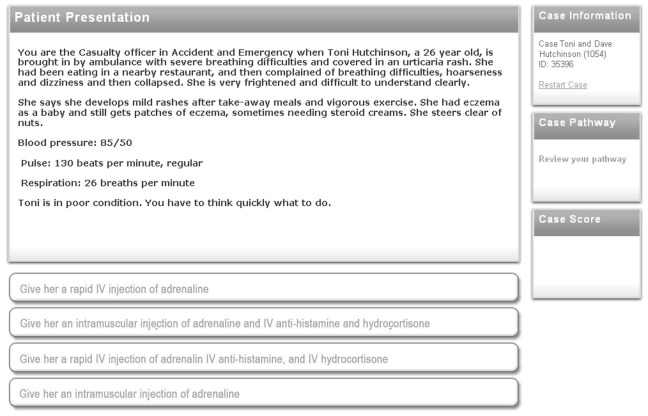
An example of a D-PBL page seen by the student PBL group at a decision point in a branching case in the authoring application OpenLabyrinth [24].

The faculty committee for each module reviewed the completed D-PBL cases to make sure that the modified cases still fell within the range of the existing learning objectives (options were based on management issues that were already described in the learning objectives). Then the linear PBL cases were supplemented by trigger questions that would cover the same area of learning emphasized by the options. For example, in the anaphylaxis case shown in Figure 1, which focused on problems caused by a rapid intravenous injection of adrenaline. The 4 options would appear only after the students had read and discussed the text (which has been shortened in this figure, for reasons of clarity). This scenario, based on a real-life incident, describes a patient presenting in Accident and Emergency with anaphylactic shock. The scenario is written to elevate stress (“Toni is in poor condition, you have to think quickly what to do”), which in turn can lead them to follow their instinctive response to give a rapid intravenous injection, as happened in real life. This proved fatal. Students have previously noted that serious consequences such as this are particularly memorable [5].

In this way, the study sought to ensure that the D-PBL provided different learning opportunities rather than additional learning opportunities. The development of the D-PBL cases has been discussed more fully elsewhere [5], although the additional prompts were not included in the linear PBL in the earlier study. Students agreed that the group would take a majority view on their chosen direction through the case.

A preliminary analysis of D-PBL in 2007 compared student engagement between learners in paired cohorts who had either a linear version of the case (without options) or a branched version (with optional routes through the case; D-PBL). Cohorts alternated between branched and linear over the first 4 weeks, with a fifth case delivered in the branched version to both cohorts. It was noted that students had performed markedly better than in previous years to a question that was related to 1 of the D-PBL decision points in the week 5 case. However, the D-PBL deployment was not designed as an experiment and there was no control group or any other means to systematically assess the impact of using D-PBL on summative assessment in this first year of using D-PBL. Therefore, a study was developed and run in the following academic year (2008) analyzing the exam performance of students with questions that had been previously encountered in either a D-PBL or linear PBL format.

### Study Design

All 81 students (50 male and 31 female) in the graduate entry class in 2008 were entered in to the study. The SGUL Teaching and Learning Committee reviewed the study protocol, noting that the study did not require further ethical review because the early test of the system had not raised any serious concerns and the intervention was restricted to relatively small changes in the execution of PBL sessions.

**Figure 2:**
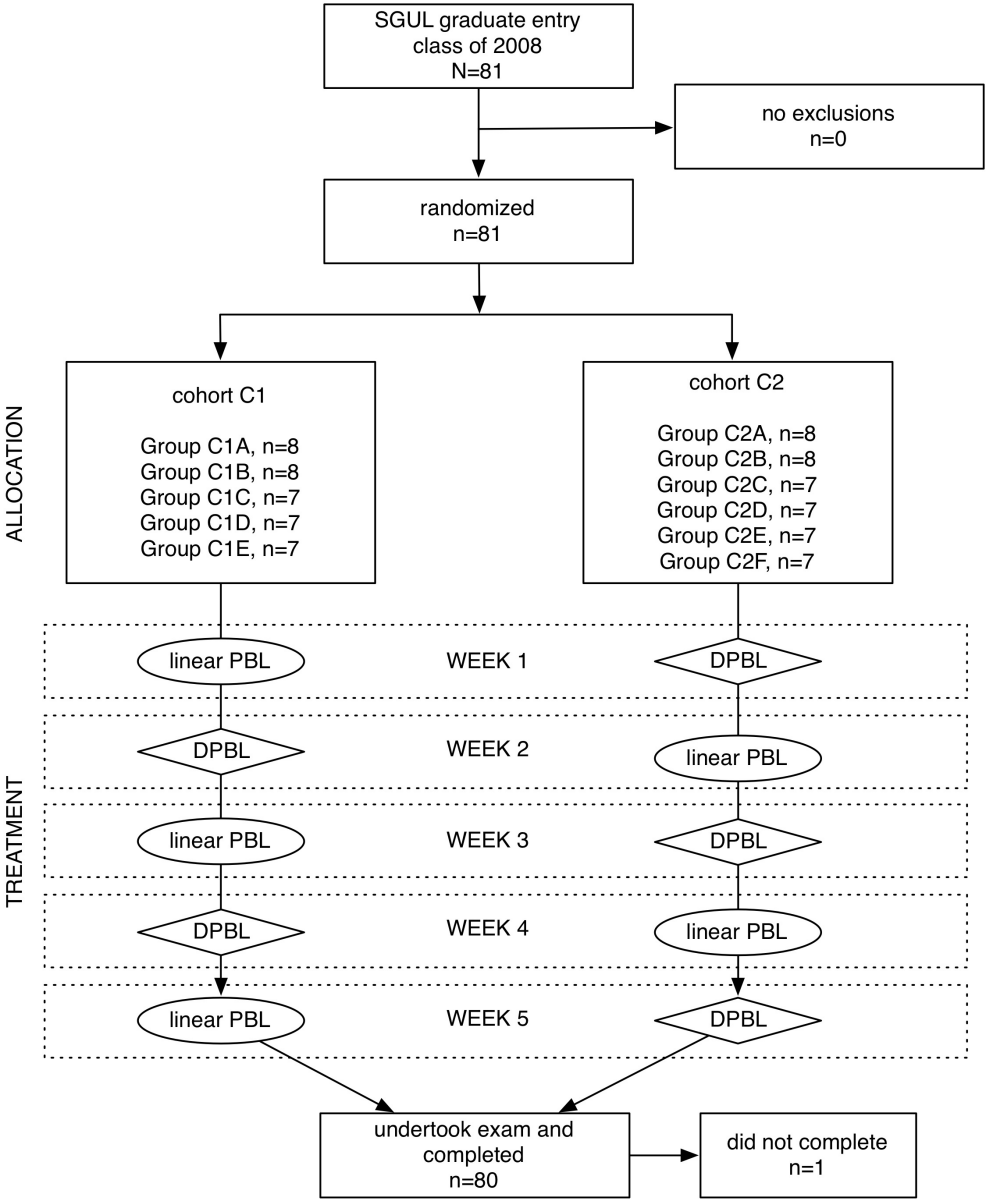
CONSORT flow diagram for the trial showing the distribution of students within the cohorts and a flow diagram of their progress through the 5 weeks of the controlled trial.

Students were randomly allocated to 11 tutorial groups (4 groups of 8 and 7 groups of 7) by the SGUL Registry as part of its standard protocol for student assignments. The 11 PBL groups were separated into 2 cohorts: C1 with 5 PBL groups and C2 with 6 PBL groups. During weeks 1, 3, and 5, cohort C1 ran linear PBL cases and C2 ran the branching D-PBL cases. During weeks 2 and 4, C1 ran branching D-PBL cases and C2 ran linear PBL cases. Blinding to the intervention was not possible; both the participants and the investigators were aware which style of case they received. The material in both groups of questions was encountered in both D-PBL and linear PBL cases. The difference was that D-PBL cases required a learner to select a choice that may have had negative consequences, whereas there were no alternative paths or consequences in the linear PBL case. The flow of participants through the study is illustrated in the Consolidated Standards of Reporting Trials (CONSORT) diagram in Figure 2.

Two weeks after the closure of the Life Protection module, the students were given a Short-Answer Question Summative Module Exam. Of the 81 students in the study, 80 took the face-to-face examination. Within the short-answer questions section of the exam, 5 questions were chosen which were significantly linked to individual options and consequences points within the relevant D-PBL case. This represented our test set of questions. Another 5 questions were deemed to have no relevance to options within the cases, and these represented a comparison set. Questions were not identified to the students as relating to the study or D-PBL within the exam. For each question, the relevant subject specialist submitted criteria for assessment and a marking scheme to the module team for review. The subject specialist had no role in the creation of the relevant option point. The module team reviewed each item and then estimated the proportion of minimally competent examinees that would correctly answer the item.

Scripts were marked in each case by the relevant subject specialist and reviewed by another marker. Maximum points for each SAQ varied from 10 to 12 marks. The Module Organizing Team ratified the validity of these 2 sets of questions and rejected 1 question for which the study team agreed did not fit cleanly into either of the 2 question categories. This reduced the comparison set to 4 questions. The examination office passed the final results to the study team who added tags to indicate which groups and cohorts they were in. The mapping between the type of PBL case, the exam questions, and the 2 cohorts are shown in Table 1.

Because the exam results for the 9 questions were found to follow a nonnormal distribution, statistical significance was tested comparing the results for each question between the D-PBL and linear PBL cohorts as independent samples employing a Mann-Whitney *U* test using SPSS v21 (IBM Corp, Armonk, NY, USA). Tests were run to evaluate these hypotheses: (1) learners who had not encountered key material related to D-PBL decisions would score lower, on average, than those who had and (2) learners who had worked with D-PBL would score the same, on average, than those who had not on material not related to D-PBL decisions. Effect sizes were calculated for the 2 cohorts using Cohen’s *d*.

**Table 1:** Exam questions used in the study mapped to the problem-based learning (PBL) case week in the Life Protection module and to the 2 cohorts’ PBL modality (linear or decision-PBL; D-PBL) in each week of the module demonstrating a spread of questions over time and between cohorts.

Week	PBL modality		Questions		Topic
	Cohort C1	Cohort C2	Related to D-PBL activity	Unrelated to D-PBL activity	
1	Linear PBL	D-PBL	0	1	Leukemia
2	D-PBL	Linear PBL	1	2	Bacterial infection
3	Linear PBL	D-PBL	1	0	Viral infection
4	D-PBL	Linear PBL	2	1	Solid tumors
5	Linear PBL	D-PBL	1	0	Allergies

## Results

**Figure 3:**
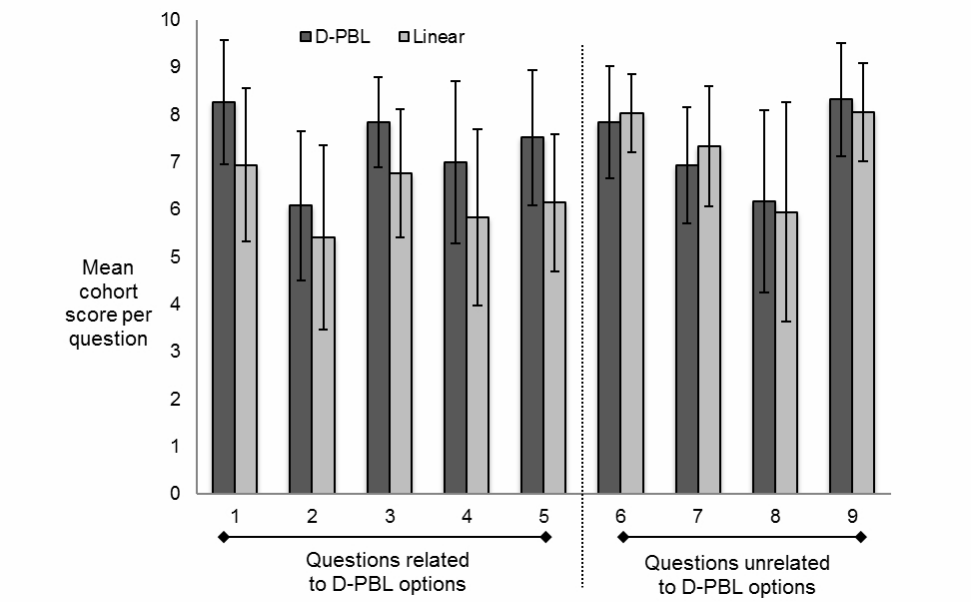
Exam question results analysis. For questions related to D-PBL option points, in each case students who had been part of the D-PBL cohort for that week performed statistically significantly better than those who were part of the linear PBL cohort. For questions unrelated to option points, there was no significant difference between the 2 groups.

The mean scores for the D-PBL cohort’s results were found to be consistently higher than for linear PBL cohort for those questions linked to D-PBL activities. The mean scores for the D-PBL cohort’s results showed no consistent pattern relative to the linear PBL cohort for those questions not linked to D-PBL choice-discussion activities. The results of the statistical analyses are shown in Table 2. Questions 1-5 were related to D-PBL and all showed a statistically significant higher level of performance for the D-PBL cohort over the linear PBL cohort. Questions 6-9 were not related to D-PBL and showed no statistically significant differences in performance between the 2 cohorts. Note *P*<.05 rejects the null hypothesis. The 80 learners in 2 cohorts were exposed to either a branching case D-PBL or linear PBL case each week (Figure 3). Each learner’s performance in the-end-of-module assessment was tracked back to which cohort they had been part of in each week. This exam contained questions that were related to the option points and questions that were unrelated to option points.

The differences between the exam performance of the D-PBL and linear PBL groups were found to be statistically significant for all questions related to D-PBL. The differences between exam performances of the 2 groups were not found to be statistically significant for any of the questions not related to D-PBL. The effect sizes for D-PBL–related questions were all large (>0.6) and positive except for question 2, which had a medium effect size (0.6-0.3) and was positive. The effect sizes for the questions not related to D-PBL were all small (<0.3) except for question 8, which had a medium effect size. Overall, there was a mix of positive and negative values.

**Table 2:** Exam question results analysis.

Question		D-PBL (n=37)		Linear PBL (n=43)		*U*	*Z*	*P* ^a^	Effect size
		Mean (SD)	Median (range)	Mean (SD)	Median (range)				
**Related to D-PBL**									
	1	8.26 (1.31)	8.50 (5.50-10.00)	6.94 (1.62)	7.00 (3.50-10.00)	443.0	–3.419	<.001	0.814
	2	6.08 (1.58)	6.00 (2.00-10.00)	5.41 (1.94)	5.50 (1.00-10.00)	593.5	–1.958	.02	0.345
	3	7.84 (0.95)	8.00 (5.00-9.50)	6.76 (1.35)	7.00 (4.00-10.00)	391.5	–3.929	<.001	0.800
	4	7.00 (1.72)	7.50 (3.00-10.00)	5.83 (1.86)	6.00 (0.50-10.00)	497.0	–2.895	.002	0.629
	5	7.52 (1.42)	7.50 (4.30-9.50)	6.15 (1.45)	6.30 (2.30-9.30)	394.0	–3.882	<.001	0.945
**Unrelated to D-PBL**									
	6	7.84 (1.18)	8.00 (3.00-9.00)	8.04 (0.82)	8.00 (6.00-9.00)	751.5	–0.437	.33	–0.244
	7	6.94 (1.23)	7.50 (4.00-10.00)	7.34 (1.27)	7.00 (2.00-9.50)	677.5	–1.153	.12	–0.315
	8	6.18 (1.92)	6.50 (0.00-9.00)	5.95 (2.31)	6.0 (1.00-10.00)	770.5	–0.242	.41	0.099
	9	8.12 (1.20)	8.00 5.00-10.00)	8.06 (1.04)	8.00 (5.00-10.00)	729.5	–0.644	.26	0.058

^a^ 1-tailed, exact.

## Discussion

### Principal Results

The results confirmed the study hypothesis that learner performance was higher for those who had learned with D-PBL than for those who had learned with linear PBL on questions related to D-PBL experiences and there were no significant differences between D-PBL and linear PBL cohort performance on questions not related to D-PBL experiences. This suggests that the use of D-PBL leads to better midterm learning outcomes than linear PBL. However, we should clarify a number of contextual factors that underpin this assertion.

Firstly, participants were midstage undergraduate learners with some limited clinical exposure. Our background hypothesis was that the D-PBL approach suited learners who already have some PBL experience and who had enough clinical knowledge to work with the patient management challenges that D-PBL involves. Secondly, it should be made clear that the intervention was the whole activity, involving the use of particular artifacts (branching virtual patient cases) in a particular way (facilitated debate and discussion at decision points) within an otherwise traditional small-group PBL setting. Research is continuing into the conduct of D-PBL activities and the experiences of those involved. It is also important to note that the Web-based D-PBL did not detract from the process of discussion, enquiry, and problem solving in PBL. Options only appeared after the preceding steps in the case had been discussed fully. Moreover, there was no need for students to find the “correct” path through a case (or to be guided in doing so) because all paths were designed to engender equivalent learning opportunities and the scenario itself provided excellent feedback in the context of the patient. If the students take poor options, the changes in the scenario are sufficient guidance to encourage students to review their choices and re-evaluate new optional routes for continuation. Therefore, careful scenario construction and testing were essential and all case writers were trained with a formal set of guidelines on scenario construction.

Because of the specificity of the activity, the type of virtual patient used within it, and the provisional nature of these current findings, no assertions can be made regarding the efficacy of virtual patients in general. We acknowledge the importance of the activity that is constructed around a virtual patient [24], something that has yet to be substantially explored [25]. This makes it somewhat difficult to compare our findings with other studies. For instance, although Nalesnik et al [26] found that exam results for groups that had PBL compared with no PBL failed to show significant efficacy for the PBL intervention, this study found little to indicate that different kinds of PBL components are more efficacious in ways that are reflected in student performance. However, D-PBL was designed for students who already had some experience with traditional linear PBL and who had acquired sufficient knowledge and expertise to be able to tackle patient management problems and deal with the consequences of their decisions. Therefore, we propose D-PBL as a modality suited to intermediate learners and this will be explored further in subsequent studies.

### Limitations

There are a number of limitations to this study. Firstly, only the 1 class iteration has been tested. Although these findings are considered strong enough to justify publication at this stage, at least to encourage others to explore the D-PBL approach, further replication studies are planned to test the efficacy of D-PBL using a wider range of learning and curriculum contexts. Secondly, an experimental approach was taken to investigate the efficacy of the D-PBL activity at the single-item level. Although this has proved useful, there is much work to be done in exploring the nature of the activity and the ways in which students experience it. Finally, it is acknowledged that the cognitive aspects of D-PBL and the links between students’ actions in small-group settings have not been explored. The nature of learning that follows from these actions and its retention and application also need further exploration.

### Comparison With Prior Work

Although our work is a hybrid of PBL and virtual patient instructional models, there are connections with studies that explore the importance of scaffolding and learner agency in PBL [27] as well as the role of collaboration and facilitation in learning [28]. The role of structured debate and the connection to clinical decision making within PBL is a departure and although there is an extant literature on decision making in medical education [29] and some consideration of the use of virtual patients to teach decision making [30], the combination of PBL, virtual patients, and clinical decision making would seem to be a significant innovation, one that shows great potential to enhance the efficacy of medical education. Although it has been proposed that virtual patients “should be designed and used to promote clinical reasoning skills” [25], we have demonstrated the efficacy (on the single-item level) of using virtual patients that require learners to engage in clinical reasoning and decision making only within a PBL activity context. We continue to explore the dynamics of D-PBL and its dependence on particular instructional contexts. D-PBL also exemplifies the importance of considering medical educational technologies in the context of the activities within which they are used [31]. Although D-PBL was only made possible by using Web-based virtual patients, much of the value of the activity was realized in the interactions between learners. The log data from the use of the D-PBL virtual patients has not yet been explored although it is expected to provide a rich area for future exploration [32]. Clearly, D-PBL has much potential as an emerging technology-enabled learning activity type.

Before this study was completed, SGUL decided to implement D-PBL in both its graduate and undergraduate (school-leaver) curricula in the common transitional year between campus-based learning and clinical attachments. This decision was based solely on tutor and student feedback. D-PBL is now running in both SGUL and the University of Nicosia, Cyprus (UNic), and in 2015 it will be implemented in the curricula of 6 further institutions in the ePBLnet consortium, a European Commission-funded program [33] implementing SGUL-style PBL. This will broaden the opportunity for further studies exploring the impact of D-PBL.

With successful implementation of the D-PBL model, our attention has turned to where the decision-making capabilities of virtual patients in PBL can be further improved. There are 3 Web-based developments in this issue that describe alternative approaches, each of which has the potential to add to the model we describe here. Kononowicz et al [34] described a method that extends the options and consequences model by permitting students to make management choices and then augmenting the interactivity of virtual patients with computational models of physiological and pathological processes. The study proposes a conceptual framework for the integration of computational models within virtual patients, discusses pilot implementations of this approach, and considers critical factors in integrating systems in this way.

Antoniou et al [35] considered the ways in which multimedia-rich 3-dimensional multi-user virtual environments (MUVE) may provide more authentic and immersive experiences for learners. The study considers the suitability of the Second Life MUVE as a virtual patient deployment platform for undergraduate dental education and explores the challenges for the successful repurposing of virtual patients from the Web to the MUVE, including case complexity, decreased textual narration, and allowing learners to go beyond narrative questions and answers.

Salminen et al [36] took a different direction and focused more on reflective practice and communication training. Rather than being based on a series of options, their model features more open-ended questions allowing free-text answers rather than the branched options used in this study.

All 3 of these interventions have been received favorably by students. The model we have described in this study has relatively low resource implications and further work is needed to establish which interventions can be widely introduced to achieve improved pedagogic value at reasonable cost.

### Conclusions

This study investigated the efficacy of D-PBL, a variant form of Web-based PBL that replaced linear PBL cases with branched virtual patients to present medical learners with alternative patient management decisions and having made a decision to deal with the consequences. Learners were encouraged to consider and discuss courses of action before taking them and to explore the consequences of their actions once they had been taken. Efficacy was measured in exam performance after a semester of weekly D-PBL sessions. It was found that D-PBL led to statistically significant improvement in student performance on key questions linked to the D-PBL process.

If the promise of our findings are borne out and D-PBL proves to be a more efficacious way of structuring learning, at least for students who have already had a year or more of traditional PBL, then this has the possibility of being a major contribution to medical education. Although our findings are provisional pending further studies in and around the D-PBL model, we propose Web-based D-PBL as a candidate activity model for improving medical education through the inclusion of structured debate and decision making in small-group learning.

## Abbreviations

D-PBL: decision–problem-based learning
MUVE: multi-user virtual environments
PBL: problem-based learning
SGUL: St George’s, University of London
